# CSMOT: Make One-Shot Multi-Object Tracking in Crowded Scenes Great Again †

**DOI:** 10.3390/s23073782

**Published:** 2023-04-06

**Authors:** Haoxiong Hou, Chao Shen, Ximing Zhang, Wei Gao

**Affiliations:** 1 Xi’an Institute of Optics and Precision Mechanics, Chinese Academy of Sciences, Xi’an 710119, China; 2 University of Chinese Academy of Sciences, Beijing 101408, China

**Keywords:** one-shot, multi-object tracking, re-ID, coordinate attention, angle-center loss, data association

## Abstract

The current popular one-shot multi-object tracking (MOT) algorithms are dominated by the joint detection and embedding paradigm, which have high inference speeds and accuracy, but their tracking performance is unstable in crowded scenes. Not only does the detection branch have difficulty in obtaining the accurate object position, but the ambiguous appearance of features extracted by the re-identification (re-ID) branch also leads to identity switches. Focusing on the above problems, this paper proposes a more robust MOT algorithm, named CSMOT, based on FairMOT. First, on the basis of the encoder–decoder network, a coordinate attention module is designed to enhance the information interaction between channels (horizontal and vertical coordinates), which improves its object-detection abilities. Then, an angle-center loss that effectively maximizes intra-class similarity is proposed to optimize the re-ID branch, and the extracted re-ID features are made more discriminative. We further redesign the re-ID feature dimension to balance the detection and re-ID tasks. Finally, a simple and effective data association mechanism is introduced, which associates each detection instead of just the high-score detections during the tracking process. The experimental results show that our one-shot MOT algorithm achieves excellent tracking performance on multiple public datasets and can be effectively applied to crowded scenes. In particular, CSMOT decreases the number of ID switches by 11.8% and 33.8% on the MOT16 and MOT17 test datasets, respectively, compared to the baseline.

## 1. Introduction

As one of the most popular high-level computer vision tasks, multi-object tracking (MOT) is widely used in the fields of autonomous driving, video surveillance, and even epidemic prevention. The objective of MOT is to locate multiple objects and obtain trajectories in a video while assigning a unique and long-term valid ID number to each object. In crowded scenes, it is highly challenging to solve the tracking problems caused by the changing appearance of the object itself and by frequent occlusions between objects.

Most of the current advanced MOT algorithms adopt a strategy based on the separating detection and embedding (SDE) paradigm. First, a detector module is used to localize objects of interest for each frame. Second, the detection results are cut out according to the bounding box and input to the embedding module to estimate the re-ID features. Finally, using the motion information and re-ID features, the detection and one of the existing tracks are matched, or a new track is created if it fails. The progress of the two abovementioned independent modules can effectively improve the overall tracking accuracy. However, since the extracted features cannot be shared between the two modules, it leads to the consumption of storage and computing resources. In crowded scenes with huge numbers of objects, in particular, the real-time performance of MOT drops sharply.

**Figure 1:**
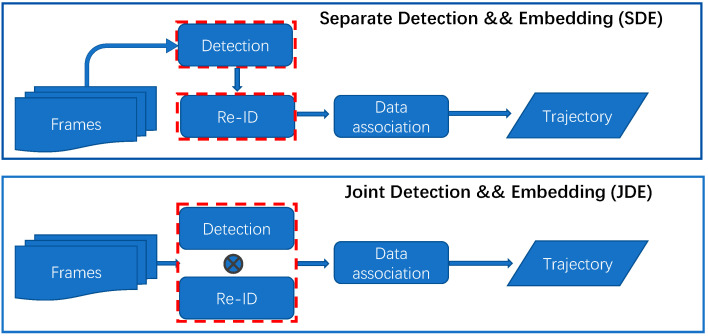
Overview of MOT algorithms with the separate detection and embedding (SDE) paradigm and the joint detection and embedding (JDE) paradigm.

In response to the complex tracking model and slow inference speed, one-shot MOT algorithms based on the joint detection and embedding (JDE) paradigm have achieved good results recently. By adding an embedding branch to the object detection network for extracting the re-ID features of objects, multi-task learning can be accomplished in a single neural network. JDE achieves end-to-end training and inferencing with real-time speed. The detection branch directly determines the tracking performance. Most JDE tracking algorithms are based on anchor-based detection networks, which are similar to Track-RCNN [1] and JDE [2]. Track-RCNN adds a fully connected layer at the head of the Mask-RCNN [3] network to extract re-ID for subsequent tracking association, enabling object tracking at the pixel level. It is still slow because Track-RCNN is an extension of the two-stage object detection algorithm. To solve this problem, Wang et al. proposed the JDE algorithm. By extending the one-stage object detection network named YOLOv3 [4], the two tasks of object detection and re-ID are completed in parallel. It is the first real-time algorithm; however, one issue is that the re-ID feature cannot be aligned with the object center, resulting in a large number of identity switches (IDs). Yifu Zhang et al. proposed FairMOT [5], based on the anchor-free object detection network CenterNet [6] and the JDE paradigm. The algorithm verifies that the anchors are not suitable for extracting re-ID features when dealing with the MOT task. FairMOT extracts high-resolution features to more effectively localize center points. The conflict between detection and re-ID is alleviated by fusing deep and shallow features. Tracking accuracy is improved, and the real-time requirement is achieved. The overview of MOT algorithms with the SDE paradigm and the JDE paradigm are shown in Figure 1.

In crowded scenes, FairMOT still suffers from a noticeable degradation of tracking performance; not only is the object detection accuracy insufficient, but the extracted re-ID features are also not discriminative enough. In this paper, we identify four factors behind this unstable performance. The first issue is caused by the complicated encoder–decoder network. Deep layer aggregation (DLA) [7] architecture can extract high-resolution features well, but densely interleaved and interconnected characteristics can also lead to information redundancy. While obtaining a larger global view, the high-frequency fusion inside the component block and across different scale layers weakens the perception of focal features and affects the accuracy of the object detection. The second issue is caused by the loss function for the re-ID branch. The re-ID branch uses the Softmax Loss function, which can better distinguish different categories of objects with significant appearance differences. However, the MOT task often needs to distinguish different objects of the same category, which are largely similar. Training with the Softmax Loss function results in the extracted re-ID features of the same object not being compact enough. The third issue is caused by re-ID features. There is a strong coupling between two different subtasks that extract features using the same encoder–decoder network. After enhancing the re-ID branch, changes in the feature dimension significantly affect the object detection performance, which in turn interferes with the overall performance of the MOT algorithm. The fourth issue is caused by data association. Regarding the prediction and matching process, previous works have only considered detection boxes with high scores. It is unreasonable to roughly filter out the low-score detection boxes output by the detection branch. On the one hand, it causes information loss and interrupts the trajectory. On the other hand, it means that the object tracking performance is heavily dependent on the detection task.

In this work, we present a simple approach, referred to as CSMOT, which elegantly addresses the four issues described above. CSMOT is built on top of CenterNet, using Fair-MOT as a baseline. Similarly, detection and re-ID tasks are integrated into one neural network. We argue that it is critical for anchor-free detection networks to extract more accurate keypoint features. The attention mechanism enables CNNs to focus on local features of interest. In this paper, we design a coordinate attention module (CAM) with very low computational and storage consumption. This module computes attention masks for high-resolution features on channels, for horizontal and vertical coordinates, enhancing its ability to locate the centers of objects. In addition, discriminative re-ID features help to solve the problem of high IDs in crowded scenes. We normalize re-ID features and corresponding fully connected layer weights so that the learning of the re-ID branch is transformed from a Euclidean space to an angular space. This transformation is inherently consistent with the mechanism for trajectory association using cosine similarity. In the angular space, we propose the angle-center loss (ACL) to increase the compactness of intra-class objects. Features from the same classes are clustered together on the surface of the hypersphere. To balance the detection and re-ID tasks, CSMOT learns relatively low-dimensional re-ID features. Our experiment demonstrates that low-dimensional features can effectively alleviate the conflict between two tasks and help to improve tracking performance. Finally, we form the effective tracking pipeline in CSMOT. Using detection boxes from high scores to low ones raises the upper bound of data association. We predict the positions of objects in the next frame with motion information and simultaneously compute the similarity of detections and tracks jointly with the IoU and re-ID features.

The main contributions of this work are three-fold: (1) an anchor-free joint detection and embedding MOT algorithm is presented, combined with the coordinate attention module. The algorithm referred to as CSMOT achieves a higher detection performance compared to the baseline method. (2) Angle-center loss optimization in angular space is proposed to supervise the re-ID branch. By setting a cosine distance penalty, maximizing intra-class similarity produces higher-quality extracted re-ID features. In addition, the feature dimension is adjusted to better balance the detection task and the re-ID task. (3) A high-performance MOT tracker is further developed by incorporating the proposed algorithm into an efficient data association strategy, which associates each detection box to avoid fragmented trajectories.

**Figure 2:**
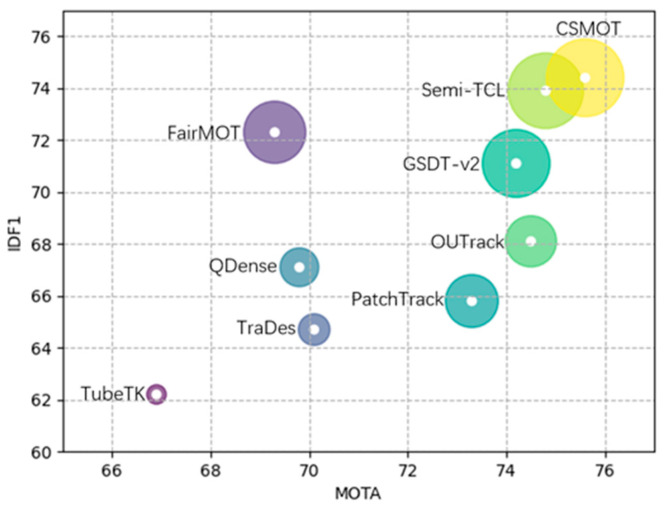
MOTA–IDF1–HOTA comparisons of different MOT algorithms on the MOT16 test set. The horizontal axis is MOTA, the vertical axis is IDF1, and the radius of the circle is HOTA. Our CSMOT outperforms the previous state-of-the-art algorithms.

Extensive experimental evaluations and analyses of the MOT benchmark demonstrate the effectiveness of CSMOT, as shown in Figure 2.

## 2. Related Work

As we know from previous works, object detection, re-ID, and data association are the three key components of multi-object tracking. We systematically optimize these three components.

### 2.1. Attention Mechanisms for Object Detection

The application of the attention mechanism in computer vision tasks enables the model to focus more on valuable information and helps to extract the features of interest. Hu et al. [8] proposed SENet, which can learn the weights of feature channels and allows important channels to obtain higher weights while suppressing the effect of less important channels. Wang et al. [9] proposed ECA-Net by using one-dimensional convolution with an adaptive kernel size to replace the fully connected layer in SENet; they obtained high-performance improvement while increasing only a very small number of parameters. However, SE and ECA attention only considers encoding inter-channel information and neglects the importance of positional information. Improved works, such as BAM [10] and CBAM [11], compress and reweight features in both the channel dimension and the spatial dimension, obtaining excellent results in multiple computer vision tasks. However, the convolutions taken by BAM or CBAM attention can only capture local relations and fail in modeling long-range dependencies.

### 2.2. Loss Function from Deep Face Recognition to re-ID

It is interesting to migrate the loss function from the field of face recognition to a re-ID subtask in MOT. Essentially, these are all fine-grained classification tasks. It is worth noting that, in our work, this is not a simple combination but a new loss function with targeted improvements. The traditional Softmax Loss (SL) continuously improves the probability of accurate classification by optimizing the error between the prediction and label. However, the features learned under SL supervision are not discriminative enough. Improvements for SL are mainly divided into two approaches: one concerns variants of SL, such as Normface [12], CosFace [13], and ArcFace [14], which make training more focused on optimizing feature mappings and feature vectors, but it is difficult to tune the parameters, and the convergence is slow during training. The other approach adds constraint items to supervise together with SL, such as Center Loss [15] and Island Loss [16]. These works are based on reducing the intra-class distance and increasing the inter-class distance to improve feature discrimination, which is highly beneficial for the MOT task.

### 2.3. Data Association

Data association is the final step of MOT, which establishes the correspondence be-tween trajectories and detection boxes. Sort [17] first uses the Kalman Filter [18] to predict the future positions of the trajectories and then calculates their overlap with the predicted boxes and uses the Hungarian algorithm [19] to assign detection boxes to trajectories. The IOU-Tracker [20] directly calculates the overlap of object detection boxes between two adjacent frames, without using the Kalman filter to predict future positions. However, they may fail when faced with the challenges of crowded scenes and fast motion. To perform data association more accurately, DeepSort [21] proposes cutting out object boxes and feeding them to the re-ID network [22] to extract appearance features. Then, it combines the location, motion, and appearance to compute the similarity between trajectories and detection boxes and uses the Hungarian algorithm to complete the optimal assignment. The method is effective in long-range matching. The above methods only select object detection boxes with high scores in the association process, which causes information loss by discarding trajectories or boxes with low scores.

## 3. CSMOT

**Figure 3:**
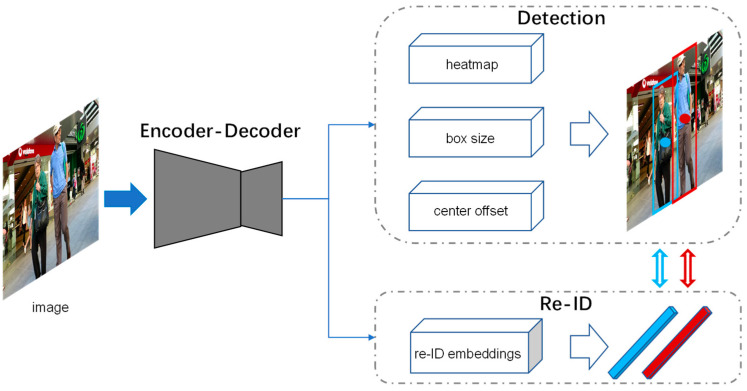
Overview of the one-shot tracker CSMOT.

In this section, we present the technical details of CSMOT, including the encoder–decoder network, the re-ID branch, and multi-task training, as well as data association. An overview of our one-shot tracker CSMOT is shown in Figure 3.

### 3.1. Encoder–Decoder Network

**Figure 4:**
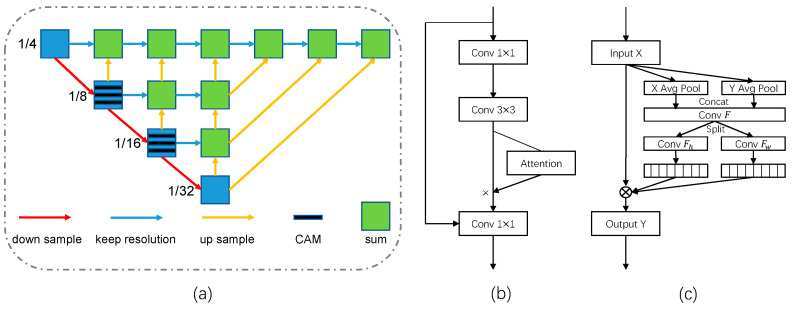
Overview of our one-shot tracker CSMOT. (**a**) Architecture of the encoder–decoder network. (**b**) Structure of the proposed coordinate attention module (CAM). (**c**) Flow of coordinate attention.

Inspired by FairMOT, our encoder–decoder network adopts ResNet-34 as the backbone and a modified deep layer aggregation (DLA) [7] for feature fusion, as shown in Figure 4a. The network has more frequent skip connections between low-level and high-level features to expand the receptive field. In addition, the deformable convolution is introduced in the up-sampling stage to dynamically adapt to changes in object scales and to enhance the generalization ability of the network in crowded scenes. Notably, the output feature map has a resolution of 1/4 as high as the original image, which facilitates the identification of small objects.

**Coordinate Attention Module (CAM).** Coordinate attention [23] is lightweight, has a high efficiency, and achieves excellent results in the fields of image classification and segmentation. Focusing on the information redundancy problem caused by dense connections in the encoder–decoder network, we designed the coordinate attention module (CAM), the structure of which is shown in Figure 4b, to extract local features of interest and improve the localization ability of object centers. During the 1/8 and 1/16 resolution stages, CAM encodes channel relationships and spatial locations, respectively. It can suppress background noise while increasing the weights of salient regions.

The CAM embeds coordinate information for the feature map. Specifically, the input feature map X has the shape of C × H × W. We encode each channel along the horizontal and vertical directions using average pooling. The coordinate information embedding process is shown in Equations (1) and (2):

(1) $$z_{c}^{h}(h)=\frac{1}{W}\sum_{0\leq i<W}x_{c}(h,i)$$

(2) $$z_{c}^{w}(w)=\frac{1}{H}\sum_{0\leq j<H}x_{c}(j,w)$$

where $z_{c}$ is the output feature map in the c-th channel. The above two transformations can integrate features along two spatial directions and generate a pair of direction-aware feature maps. The CAM captures long-range interactions spatially by incorporating information in the horizontal and vertical directions.

The features in two spatial directions are concatenated to ensure that the outputs have a consistent dimension, and they are next fed into a shared 1 × 1 convolution, as shown in Equation (3):

(3) $$f=F^{1\times 1}(cat(z^{h},z^{w}))$$

where $F^{1\times 1}$ is a convolutional transformation function, cat is the concatenation operation, and $z^{h}$ and $z^{w}$ denote the features extracted by Equations (1) and (2), respectively. Then, we split the feature map f into two separate tensors, $f^{h}$ and $f^{w}$, in the spatial dimension. Finally, two other convolutions, $F_{h}^{1\times 1}$ and $F_{w}^{1\times 1}$, are used to transform $f^{h}$ and $f^{w}$ to tensors with the same channel number as the input X, which are shown in Equations (4) and (5):

(4) $$g^{h}=F_{h}^{1\times 1}(f^{h})$$

(5) $$g^{w}=F_{w}^{1\times 1}(f^{w})$$

The outputs $g^{h}$ and $g^{w}$ represent the attention weights of the coordinates in the X and Y directions, respectively, and the final output of CAM is shown in Equation (6):

(6) $$y_{c}(i,j)=x_{c}(i,j)\times g_{c}^{h}(i)\times g_{c}^{w}(j)$$

where $x_{c}(i,j)$ and $y_{c}(i,j)$ denote feature values with the coordinates (i,j) and the c-th channel in the input and output feature maps. The whole flow is shown in Figure 4c.

### 3.2. Re-ID Branch

The role of the re-ID branch is to generate features that can recognize different objects. For multi-object tracking tasks, object similarity matching is a fine-grained process. Different objects of the same category have high similarity. In crowded scenes, frequent inter-object interactions and non-object occlusions create higher requirements for the discriminativeness of re-ID features [24].

**Angle-Center Loss (ACL).** In this paper, we propose the angle-center loss (ACL) for supervising the re-ID branch. This comprises two main parts. Our approach normalizes the feature vector and the weight vector, thus projecting features from the original Euclidean space into the angular space. Based on the concept of central clustering, we set an angle-center penalty term to reduce the cosine distance within the class.

The Softmax Loss (SL), which is widely used in coarse-grained classification tasks, is defined in Equation (7):

(7) $$L_{S}=-\frac{1}{N}\sum_{i=1}^{N}\log\frac{e^{W_{y_{i}}^{T}x_{i}+b_{y_{i}}}}{\sum_{j=1}^{n}e^{W_{j}^{T}x_{i}+b_{j}}}$$

where N is the batch size of training and n is the number of classes. $x_{i}$ denotes a feature vector of the i−th sample belonging to class $y_{j}$. W and b denote the weight and bias in the last fully connected layer of the network, respectively. For the simplicity of the implementation and optimization, we set the bias b=0. Thus, the exponential term in Equation (7) can be transformed from the vector inner to the angular cosine, as shown in Equation (8):

(8) $$W^{T}x=\left\|W\right\|\left\|x\right\|\cos\theta$$

where θ denotes the angle between the weight vectors W and feature vectors x. Furthermore, we regularize the weight vectors and feature vectors with $L_{2}$ normalization. We fix $\left\|W\right\|=1$ and $\left\|x\right\|=1$. This allows the training to be more focused on optimizing the angle θ. During the MOT data association process, the similarity between two re-ID features is computed, using the cosine distance as a metric. This suggests that the norm is more firmly in line with object discrimination. The normalized SL is shown in Equation (9):

(9) $$L_{NS}=-\frac{1}{N}\sum_{i=1}^{N}\log\frac{e^{r\cos(\theta_{y_{i},i})}}{\sum_{j=1}^{n}e^{r\cos\theta (\theta_{j,i})}}$$

where r is a hyperparameter. We constrain the feature vectors to a hypersphere of the radius r by normalization.

However, the learned features driven by the $L_{NS}$ are divided only by the number of categories, ensuring that the classes are separable but not requiring intra-class compactness. This is not suitable for fine-grained classification. To improve the discriminability of the features, we propose the angle-center loss. The loss penalty term is set by calculating the cosine distance between a sample and its category center. The definition of ACL is shown in Equation (10):

(10) $$L_{AC}=\frac{1}{N}\sum_{i=1}^{N}\frac{\left(1-\cos(\bar{\theta_{c_{i},i}})\right)}{n_{c_{i}}}$$

where $c_{i}$ denotes the category feature center to which the i−th sample belongs. $\bar{\theta_{c_{i},i}}$ is the angle between feature center and the sample feature. $n_{c_{i}}$ is the number of samples belonging to $c_{i}$ in the batch. When calculating the sum of the cosine distances for each class, we divide by the number of samples in that class to obtain the mean value. This is to avoid the problem of unsynchronized gradient updates in different classes due to sample imbalance. ACL can pull all of the features of each category toward the corresponding category center. We further tighten the intra-class space while reducing the classification error by joint $L_{NS}$ and $L_{AC}$ training. The re-ID features learned are made to have the stronger representational ability. The loss function for the re-ID branch is shown in Equation (11):

(11) $$L_{re-ID}=(1-\alpha )L_{NS}+\alpha L_{AC}=-\frac{\left(1-\alpha \right)}{N}\sum_{i=1}^{N}\log\frac{e^{r\cos(\theta_{y_{i},i})}}{\sum_{j=1}^{n}e^{r\cos(\theta_{j,i})}}+\frac{\alpha }{N}\sum_{i=1}^{N}\frac{\left(1-\cos(\bar{\theta_{c_{i},i}})\right)}{n_{c_{i}}}$$

where α is used as a hyperparameter to balance the two loss functions. When α=0, $L_{re-ID}$ degenerates to $L_{NS}$.

**Re-ID Feature Dimension.** In previous re-ID works, high-dimensional features achieved good results. However, the re-ID feature dimension cannot be considered independently in MOT tasks when adopting the JDE paradigm algorithm. There is a strong coupling between the detection task and the re-ID task that share most features. Re-ID features that are too high dimensional have a negative impact on the detection task. Inaccurate detection further affects the extraction of re-ID features, eventually leading to an overall decrease in comprehensive tracking performance. More importantly, the re-ID branch should re-adapt the feature dimension to the proposed encoder–decoder network under the supervised learning of ACL. Our experiment demonstrates that learning low-dimensional re-ID features is more beneficial to both subtasks.

### 3.3. Multi-Task Training

The proposed CSMOT adopts joint-loss training for the supervised learning of both the detection and re-ID branches. For the detection branch, the heatmap head uses focal loss [25] to estimate the locations of the object centers, which can effectively deal with the problem of unbalanced samples between the center point and the surrounding points. Then, we enforce L1 loss for the box size and offset heads. Moreover, the re-ID branch uses the proposed loss as Equation (11). We dynamically balance the two branches by an uncertainty loss in Equations (12) and (13):

(12) $$L_{\det ection}=L_{heatmap}+L_{box\_size}+L_{box\_offset}$$

(13) $$L_{total}=\frac{1}{2}(\frac{1}{e^{\beta_{1}}}L_{\det ection}+\frac{1}{e^{\beta_{2}}}L_{re-ID}+\beta_{1}+\beta_{2})$$

where $\beta_{1}$ and $\beta_{2}$ are learnable parameters that balance the two branches during training. We set the initial values as −1.85 and −1.05, following FairMOT.

### 3.4. Data Association

We follow a simple and effective data association strategy in ByteTrack [26] and form the online tracking pipeline in CSMOT. Unlike the original ByteTrack, we not only use IoU but also add re-ID features in the similarity computation process.

In crowded scenes, object detection scores tend to decrease slowly with increasing occlusion. We track each detection box, not only high-score detection boxes. The similarity of low-score detection boxes and unmatched tracks can recover true objects, and false-positives are ignored. The specific association process is as follows.

(1) Step 1. Input a new frame to CSMOT and obtain the detection boxes and corresponding scores through the detection branch. Assign detection boxes with scores above threshold $T_{high}$ to group $G_{high}$, and assign those with scores between $T_{low}$ and $T_{high}$ to $G_{low}$;
(2) For all tracks in the existing trajectories T, we use the Kalman Filter to predict the new position for the next frame;
(3) The high-score detection box $G_{high}$ is associated with the predicted boxes of trajectories T. We compute the similarity using IoU and re-ID features and use the Hungarian algorithm to finish the matching. Unmatched detections and tracks are separately assigned to $G_{cache}$ and $T_{cache}$;
(4) The low-score detection box $G_{low}$ is associated with unmatched tracks of trajectories $T_{cache}$. The unmatched detection boxes are treated as the background and deleted directly. For unmatched tracks, we mark them as $T_{re-cache}$. Because low-score detections mean that the appearance features are not credible, we only use IoU to compute the similarity in this association.
(5) To implement the long-range association, we put $T_{re-cache}$ into $T_{lost}$. When unmatched tracks appear in $T_{lost}$ for more than 30 frames, we delete these tracks completely. Otherwise, we keep the lost tracks $T_{lost}$ in T.
(6) For each high-score detection in unmatched $G_{cache}$, we initialize a new track if the score exceeds the threshold τ and appears in two consecutive frames.

## 4. Experiments

### 4.1. Experimental Settings

**Datasets.** The JDE-based CSMOT proposed in this paper consists of three tasks to be learned: object detection, re-ID, and MOT. Therefore, we build a large-scale hybrid dataset for different tasks to jointly train the model following FairMOT. The joint dataset contains rich scenes and a large number of object annotations, which is conducive to improving the generalization and robustness of the MOT algorithm. Regardless of whether or not we add identity annotations during training, we divide the dataset into two categories. The first category includes CrowdHuman (CH) [27], ETH [28], and CityPersons (CP) [29]. We only use the bounding box annotations of these datasets to train the detection branch of our CSMOT. The CH contains many dense pedestrian annotations in crowded scenes, which can significantly improve the tracking ability. The second category includes CalTech (CT) [30], CUHK-SYSU (CS) [31], PRW [32], and MOT17 [33]. Bounding boxes and identity annotations provided by the category are used to train both the detection and re-ID branches. Specifically, we remove video frames in ETH that overlap with the MOT17 test set for fairness. We present ablation experiments on the validation set of MOT17 and compare the tracking ability with that of other MOT algorithms on the MOT Challenge server. The statistics of the hybrid dataset are shown in Table 1.

**Metrics.** In order to make the evaluation results more accurate and objective, we use the general MOT Challenge Benchmark metrics [32]. The metrics in this paper include false-positive (FP ↓), false-negative (FN ↓), the number of identity switches (IDs ↓), multiple-object tracking accuracy (MOTA ↑), identification F1 score (IDF1 ↑), and higher-order tracking accuracy (HOTA ↑). Here, ↑ means higher is better, and↓ means lower is better. MOTA equally considers FP, FN, and IDs in the trajectory. Since the number of FPs and FNs is much larger than that of IDs, MOTA is more inclined to measure the detection performance. IDF1 focuses on whether the ID of the track remains the same throughout the tracking process. IDF1 is more sensitive to the performance of data association. HOTA is a very recently proposed metric, which computes the geometric mean of detection accuracy and association accuracy.

**Implementation Details.** The experimental environment is a deep learning server with an Intel Xeon CPU Gold 6130 processor and two RTX 2080 Ti GPUs. We evaluate the tracking performance using a single GPU. For CSMOT, we employ DLA-34 [10] as the backbone network and initialize the algorithm model by adopting CenterNet [6] detection model parameters that have been pre-trained on the COCO [34] dataset. The input image is resized to 1088 × 608. During data preprocessing, we introduce standard data augmentation methods including rotation, scaling, and color jittering. We train our CSMOT with the Adam optimizer for 40 epochs, with an initial learning rate of 10^-4^. At the 20th epoch and 35th epoch, the learning rate decreases to 10^-5^ and 10^-6^, respectively. The model is trained with a batch size of 12. The total training time is about 40 h.

### 4.2. Ablation Studies

In this section, we present rigorous studies of the four critical factors mentioned in Section 1, including the encoder–decoder network, re-ID branch loss, feature dimensions, and data association. We train CSMOT on a combination of CrowdHuman and the MOT17 half-training set, if not specified. The remaining half of the MOT17 training set is used for validation. Additionally, we perform a fair comparison with advanced one-shot MOT algorithms and a training data ablation study.

**Encoder–Decoder Network.** This section presents the tracking performance between the unmodified DLA-34 network and those with CAMs, which are inserted at the head, neck, and backbone locations. The results are shown in Table 2.

Notably, the head represents the detection and re-ID branch in Figure 3. The neck and backbone, respectively, represent the “blue” basic blocks and the “green” aggregation blocks in Figure 4. Our experiments show that CAM is sensitive to location. The tracking performance degrades when the CAMs are inserted into the head and neck locations of the encoder–decoder network. On the one hand, because the resolution of feature maps at the head is too low, the additional spatial masks instead introduce a large proportion of non-pixel information. On the other hand, the number of channels at the neck is large, and the frequent adjustment of the relationship between channels can easily lead to overfitting. When we combine CAMs with the basic blocks in the backbone, which is responsible for feature extraction, it improves the MOTA from 71.2 to 71.9 and the IDF1 from 74.7 to 75.1 and decreases the IDs from 413 to 383. At the same time, it leads to only a small decrease in the inference speed. Therefore, CAMs are more suitable for the middle layers of the encoder–decoder network with a moderate spatial resolution and number of channels. Increasing the weight on the object center improves the tracking accuracy in crowded scenes.

**Re-ID Branch Loss.** In this section, the tracking performance is presented under the supervision of two loss functions and the proposed angle-center loss. We set the hyperparameter α in Equation (11) to 0.001. The results are shown in Table 3.

The re-ID subtask in multi-object tracking is a fine-grained classification process. Classifying objects with high similarity necessitates more stringent requirements for the re-ID features. We can see that Normed-Softmax achieves a better performance than Softmax for all metrics, which indicates that optimizing the angle θ in Equation (8) rather than the inner product can make re-ID features more discriminative. This is fully consistent with the method of using the cosine distance to compute the similarity of re-ID features. In addition, the proposed angle-center loss improves the MOTA of Normed-Softmax from 71.2 to 71.6 and the IDF1 from 74.7 to 75.6 and decreases the IDs from 413 to 365. The numbers of FPs and FNs are also minimized. The cosine distance constraint term based on the angular center can make the intra-class distance more compact. In crowded scenes, high-quality re-ID features can re-associate objects after severe occlusion.

**Re-ID Feature Dimensions.** Previous two-step MOT algorithms usually learn 512-dimensional re-ID features. High-dimensional features are effective for algorithms that use an independent network to extract re-ID features. Our experiments show that one-shot algorithms based on the joint detection and re-ID paradigm are better adapted to lower-dimensional features. The subtasks in multitask learning are coupled with each other, and the feature dimension plays an important role in balanced learning. We evaluate two choices for the re-ID feature dimensions of JDE, FairMOT, and CSMOT in Table 4.

For JDE, the 64-dimensional feature performs better than the 512-dimensional feature for all metrics. For FairMOT and CSMOT, the performance of the two algorithms is similar. We can see that 512 achieves higher IDF1 scores, which indicates that the high-dimensional re-ID features have stronger discriminability. However, 64 performs better on the MOTA and ID metrics. Lower feature dimensions can reduce the constraints on the detection branch, and more accurate detections further ensure the continuity of the trajectory. For one-shot MOT algorithms, the re-ID features can be adaptively adjusted to low dimensions to balance the two subtasks of detection and re-ID.

**Data Association Methods.** This section evaluates two ingredients, the bounding box IoU and re-ID features, in different data association methods including MOTDT [35] and ByteTrack. MOTDT integrates motion-guided box propagation results and detection results to associate unreliable detection results with tracklets. The results are shown in Table 5.

Box IoU and re-ID features are used to compute the similarity between detections and tracks. We can see that relying solely on box IoU leads to a poor tracking performance for both methods. IoU cannot cope with re-identification after severe occlusion between objects. This is particularly true for crowded scenes. Adding re-ID features significantly increases IDF1 and decreases the number of ID switches, which also improves MOTA. Accordingly, the importance of high-quality re-ID features for tracking is also confirmed. ByteTrack improves the MOTA of MOTDT from 71.8 to 72.6 and the IDF1 from 75.6 to 76.1 and decreases IDs from 348 to 289. By making full use of low-score detections to associate trajectories, it can improve tracking accuracy and reduce the rate of fragmented trajectories.

**Comparison of Advanced One-Shot MOT Algorithms.** Advanced works based on joint detection and embedding include JDE, TrackRCNN, and FairMOT. For fairness, we use the same training data to compare all of these methods, as described in the relevant papers. The test set is derived from six videos of 2DMOT15. JDE, FairMOT, and CSMOT all use the large-scale dataset described in Datasets. Since TrackRCNN requires segmentation labels for training, only four videos with segmentation labels from MOT17 were used as the training set. The results are shown in Table 6.

When the training set is the large-scale HYBRID, we achieve a significant improvement in the performance of CSMOT compared to that of JDE. The IDF1 score increases from 66.7 to 80.5, and the number of ID switches decreases from 218 to 51. This is because the anchor-free method can better solve the problem of the ambiguous expression of anchor boxes in the MOT task. Without loading pre-trained weights, CSMOT has an advantage over FairMOT in the IDF1 and IDs metrics, which proves its better performance in maintaining trajectory continuity. When the training set is the small-scale MOT17Seg, CSMOT has an overwhelming advantage over TrackRCNN and FairMOT. CSMOT achieves a much higher IDF1 score (75.9 vs. 49.4, 64.0), a higher MOTA (72.9 vs. 69.2, 70.2), and fewer ID switches (69 vs. 294, 96). This proves that the proposed CSMOT has stronger generalization and robustness on the small-scale dataset.

**Comparison of Different Training data.** We evaluated the performance of CSMOT using different combinations of training data, and the results are shown in Table 7.

When only the first half of MOT17 is used for training, a MOTA of 67.6 and an IDF1 of 69.9 are achieved. This already constitutes an outperformance of most MOT algorithms, which shows the superiority of our CSMOT. When further adding CrowdHuman, the MOTA and IDF1 metrics improve significantly. On the one hand, CrowdHuman boosts the detection branch, enabling it to recognize occluded objects. On the other hand, more accurate detection boxes can improve the performance of data association. In addition, when we add large-scale training datasets, MOTA and IDF1 achieved improvements of only 0.1 and 0.7, respectively, because the network model has already achieved good fitting under the training of the CrowdHuman and MOT17 datasets. The experimental results prove that CSMOT is not data-hungry, which is an advantage in many applications.

### 4.3. MOT Challenge Results

We compare our CSMOT to the previous state-of-the-art MOT algorithms on the test sets of MOT16 and MOT17, including the two-step methods shown in Table 8.

It is worth noting that all the results come directly from the MOT Challenge server. In particular, MOT16 and MOT17 contain rich crowded scenes. We can see that CSMOT significantly outperforms other algorithms in terms of the MOTA, IDF1, HOTA, and ID metrics. For the results obtained for the MOT17 test set, we achieved the same MOTA as the second performance algorithm, TrackFormer. However, CSMOT outperforms the second one by a large margin in terms of the other metrics (i.e., +5.5 IDF1, +2.8 HOTA, −26.2% FP, and −22.7% IDs). In addition, CSMOT outperforms FairMOT in terms of almost all metrics and decreases the number of ID switches by 33.8%. All of these findings indicate that our approach achieves a very good tracking performance.

### 4.4. Qualitative Results

**Figure 5:**
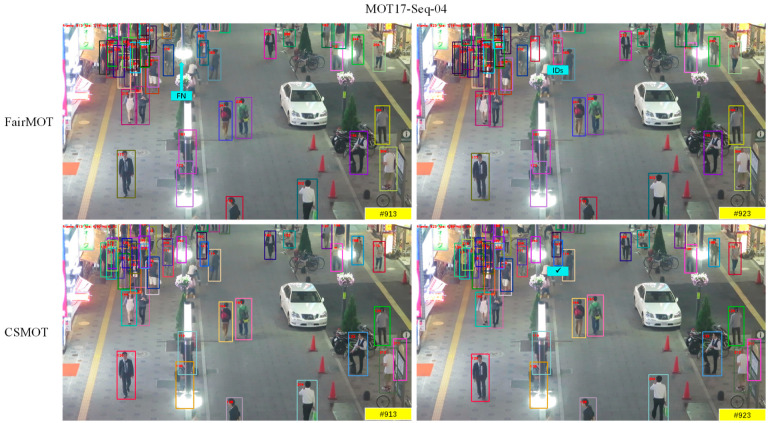

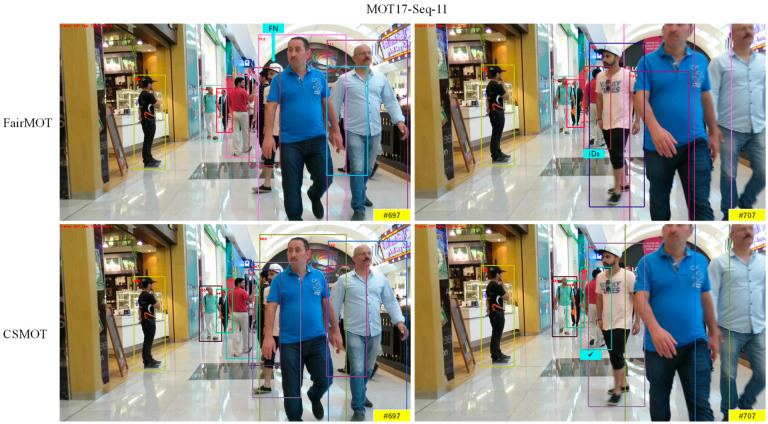
Robustness of our CSMOT compared to FairMOT. IDs and FN, respectively, indicate that the identity of the tracked object is switched and the object is not recognized. The checkmark indicates that the identity of the object is not switched.

The visualized tracking results of CSMOT compared to FairMOT on the test sets of MOT17-Seq-04 and MOT17-Seq-11 are shown in Figure 5.

We use the models with the same training datasets, CrowdHuman and the first half set of MOT17, to generate the visualization results. The difficult cases include severe occlusion (i.e., MOT17-Seq-04) and screen shake with camera motion (i.e., MOT17-Seq-11). From the results of MOT17-Sep-04, we can see that CSMOT can assign correct identities with the help of high-quality re-ID features when the objects are mostly covered up. In particular, small objects with large information loss can be detected correctly. The results of MOT17-Seq-11 show that our approach can deal with large-scale variations in crowded scenes. As we can see from the abovementioned difficult cases, our one-shot MOT algorithm achieves a significantly better tracking performance and does not lead to any identity switches in crowded scenes.

## 5. Conclusions

In this paper, we propose an enhanced one-shot MOT algorithm named CSMOT, which adopts the joint detection and embedding paradigm. A novel coordinate attention module (CAM) and angle-center loss (ACL) are proposed to improve the performance of the encoder–decoder network and the re-ID branch. Furthermore, we redesign the re-ID feature dimension to mitigate the competition between the detection and ReID subtasks. During the data association, we associate low-score detection boxes with unmatched tracks, which reduces the dependence of tracking on detection results. The experiments show that CSMOT outperforms other advanced MOT algorithms in terms of almost all metrics. In particular, our approach can significantly decrease the number of ID switches to ensure the continuity of the tracking trajectory, which is more adaptable to crowded scenes with severe occlusion. However, the current MOT algorithm has poor real-time performance and is difficult to deploy in scenarios with insufficient computing power. In the future, we will consider designing a more lightweight model to reduce storage and computing consumption.

## Figures and Tables

**Table 1:** Statistics of the hybrid dataset.

Dataset	CH	ETH	CP	CT	CS	PRW	MOT17	Total
#Img	20 K	2 K	3 K	27 K	11 K	6 K	5 K	74 K
#Box	470 K	17 K	21 K	46 K	55 K	18 K	112 K	740 K
#ID	-	-	-	0.6 K	7 K	0.5 K	0.5 K	8.7 K

**Table 2:** Comparison of the coordinate attention module (CAM) at different locations for the encoder–decoder network. Here, **↑** means higher is better, and **↓** means lower is better.The best results are shown in **bold**.

Location	MOTA ↑	IDF1 ↑	FP ↓	FN ↓	IDs ↓	FPS ↑
None	71.2	74.7	3516	11,635	413	**14.5**
Head	70.6	73.4	3523	**11,583**	389	13.6
Neck	70.8	73.2	3720	11,667	407	13.8
Backbone	**71.9**	**75.1**	**3012**	11,718	**383**	14.1

**Table 3:** Comparison of two loss functions and proposed angle-center loss (ACL) for the re-ID branch in CSMOT. The best results are shown in **bold**.

Methods	MOTA ↑	IDF1 ↑	FP ↓	FN ↓	IDs ↓
Softmax	70.0	70.3	3661	12,335	495
Normed-Softmax	71.2	74.7	3516	11,635	413
Angle-Center Loss	**71.6**	**75.6**	**3498**	**11,465**	**365**

**Table 4:** Evaluation of the re-ID feature dimensions of JDE, FairMOT, and CSMOT. The best results of the same method are shown in **bold**.

Methods	Dim	MOTA ↑	IDF1 ↑	IDs ↓
JDE	512	59.9	64.1	536
JDE	64	**60.3**	**65.0**	**474**
FairMOT	512	68.5	**73.7**	312
FairMOT	64	**69.2**	73.3	**283**
CSMOT	512	71.9	**75.4**	330
CSMOT	64	**72.5**	73.7	**323**

**Table 5:** Evaluation of the two ingredients in MOTDT and ByteTrack. The best results are shown in **bold**.

Methods	Box IoU	Re-ID	MOTA ↑	IDF1 ↑	IDs ↓
MOTDT	✓		71.6	72.3	378
	✓	✓	**71.8**	**75.6**	**348**
ByteTrack	✓		71.7	74.7	698
	✓	✓	**72.6**	**76.1**	**289**

**Table 6:** Comparison of the advanced one-shot algorithms on the 2DMOT15 validation set. “HYBRID” represents the large-scale training dataset. “MOT17Seg” stands for the four videos with segmentation labels in the MOT17 dataset. The best results of the same training data are shown in **bold**.

Training Data	Methods	MOTA↑	IDF1↑	FP↓	FN↓	IDs↓
HYBRID	JDE	67.5	66.7	1881	**2086**	218
	FairMOT	**77.2**	79.8	**757**	2094	80
	CSMOT	77.1	**80.5**	812	2140	**51**
MOT17Seg	TrackRCNN	69.2	49.4	1328	2349	294
	FairMOT	70.2	64.0	1209	2537	96
	CSMOT	**72.9**	**75.9**	**1132**	**2276**	**69**

**Table 7:** Comparison of different training data on the MOT17 validation set. “MOT17” is short for the MOT17 half-training set. “CH” is short for the CrowdHuman dataset. “HYBRID” represents the large-scale training dataset described in **Datasets**. The best results are shown in **bold**.

Training Data	Images	MOTA ↑	IDF1 ↑	IDs ↓
MOT17	2.7 K	67.6	69.9	378
CH + MOT17	22.7 K	**72.6**	**76.1**	**289**
CH + MOT17 + HYBRID	71.7 K	**72.7**	**76.8**	**258**

**Table 8:** Comparison of the state-of-the-art algorithms under the “private detector” protocol on the MOT16 and MOT17 test sets. The best results of each dataset are shown in **bold**.

Methods	Published	MOTA ↑	IDF1 ↑	HOTA ↑	FP ↓	FN ↓	IDs ↓
MOT16							
TubeTK [36]	CVPR20	66.9	62.2	50.8	11,544	47,502	1236
FairMOT [5]	IJCV21	69.3	72.3	58.3	13,501	41,653	815
QDense [37]	CVPR21	69.8	67.1	54.5	9861	44,050	1097
TraDeS [38]	CVPR21	70.1	64.7	53.2	**8091**	45,210	1144
PatchTrack [39]	CVPR22	73.3	65.8	56.9	16,092	**31,891**	1179
OUTrack [40]	NCom22	74.2	71.1	59.2	13,207	32,584	1328
GSDT-v2 [41]	ICRA21	74.5	68.1	56.6	8913	36,428	1229
Semi-TCL [42]	CVPR21	74.8	73.9	60.3	8334	36,685	925
CSMOT	Ours	**75.6**	**74.4**	**60.6**	9196	34,552	**719**
MOT17							
TransCenter [43]	CVPR21	73.2	62.2	54.5	23,112	123,738	4614
GSDT-v2 [41]	ICRA21	73.2	66.5	55.2	26,397	120,666	3891
Semi-TCL [42]	CVPR21	73.3	73.2	59.8	**22,944**	124,980	2790
OUTrack [40]	NCom22	73.5	70.2	58.7	34,731	110,586	4122
PatchTrack [39]	CVPR22	73.6	65.2	53.9	23,976	121,230	3795
FairMOT [5]	IJCV21	73.7	72.3	59.3	27,507	117,477	3303
PeTrack [44]	ICCV21	73.8	68.9	55.5	28,998	115,104	3699
TrackFormer [45]	CVPR22	74.1	68.0	57.3	34,602	**108,777**	2829
CSMOT	Ours	**74.1**	**73.5**	**60.1**	25,530	118,476	**2187**

## Data Availability

Not applicable.
